# Situational analysis of pesticide poisoning and perceptions of autoinjector devices in rural communities in Sri Lanka – a study protocol

**DOI:** 10.1080/16549716.2024.2434372

**Published:** 2024-11-29

**Authors:** Janet Perkins, Alice Street, Upul Wickramasinghe, Manjula Weerasinghe, Michael Eddleston, Jane Brandt Sørensen

**Affiliations:** a Department of Social Anthropology, School of Social and Political Science, University of Edinburgh, Scotland, UK; b South Asian Clinical Toxicology Research Collaboration, University of Peradeniya, Peradeniya, Sri Lanka; c Centre for Pesticide Suicide Prevention, and Pharmacology, Toxicology and Therapeutics, Centre for Cardiovascular Science, University of Edinburgh, Edinburgh, UK; d Department of Community Medicine, Faculty of Medicine and Allied Sciences, Rajarata University of Sri Lanka, Anuradhapura, Sri Lanka; e Global Health Section, Department of Public Health, University of Copenhagen, Copenhagen K, Denmark

**Keywords:** Pesticide poisoning, atropine, autoinjectors, implementation science, qualitative research, anthropology, Sri Lanka

## Abstract

Intentional and unintentional pesticide poisoning is an important public health problem, especially in low- and middle-income countries. Individuals who have been exposed to toxic pesticides, particularly organophosphorus insecticides, need early treatment. Atropine autoinjector devices offer a potential solution, allowing storage of effective treatment near agricultural workers’ fields and homes that could be reached within minutes by the worker or fellow villagers to provide first-line emergency care. Here we present the design of a qualitative, formative study that will constitute the first phase of an implementation science study exploring the introduction of atropine autoinjectors in rural villages.

This study will employ a qualitative design to investigate the feasibility and operational opportunities and challenges in providing pre-hospital emergency care with atropine autoinjectors in rural communities in Sri Lanka. We will conduct semi-structured interviews, ethnographic observations, oral history interviews, participatory mapping, and focus group discussions in villages and in hospitals.

This study will allow the design of an autoinjector intervention that is tailored to specific needs of rural communities, maximise the potential benefits in the villages where they are placed, and contribute to knowledge related to biomedical technologies designed for use in LMICs. It will also contribute to social science scholarship in the context of pesticide poisoning. Study approvals have been obtained from the University of Edinburgh Medical School Research Ethics Committee (23-EMREC-039) and from Ethics Review Committee, Faculty of Medicine and Allied Sciences, Rajarata University of Sri Lanka (ERC/2023/4).

## PAPER CONTEXT

- **Main findings:** Pesticide poisoning in agricultural areas in low- and middle-income countries is a crucial public health problem and atropine autoinjector devices offer a thus far unexplored potential solution to treating individuals immediately after exposure to toxic pesticides.
- **Added knowledge:** This paper presents the design of a qualitative, formative study that will constitute the first phase of an implementation science study exploring the people’s relationships to pesticides and the potential of introducing atropine autoinjectors in rural Sri Lankan villages.
- **Global health impact for policy and action:** This study will contribute valuable insights to socially informed global and national health policy addressing pesticide toxicity in low- and middle-income countries.

## Background

Pesticides, a mixture of chemical substances used to kill pests, are widely used by small-scale farmers across low- and middle-income countries (LMIC). As a result of the Green Revolution in the 1950/60s, high-yield crop varieties dependent particularly on fertilisers but also on chemical pesticides (especially insecticides and herbicides) were introduced into agriculture [1,2]. The use of these plants and chemicals has resulted in sustained increases in crop production. However, this agricultural benefit has come at marked health and environmental cost [1,3–5]. Households suddenly had access to highly hazardous pesticides but were completely unprepared to use or store them safely. As a result, more than 14 million deaths have occurred from acute occupational poisoning and intentional pesticide poisoning over the last 60 years [6].

Pesticide poisoning can result from accidental or intentional exposure. Accidental exposure includes exposure to pesticides in the environment or food [7,8], or through occupational exposure that can occur during preparation, mixing, loading and application of pesticides [9]. Research has found that there is usually very limited knowledge about the harmful impact of the many pesticides used globally on health and the environment [10–13]. Unaware of these harms, many agricultural workers, especially in LMICs, use higher doses of pesticides than recommended and without or with limited personal protection [14]. Long-term exposure can result in harm to human health, such as disturbances to the nervous, reproductive, respiratory, immune and cardiovascular systems [15]. In addition, food and water are contaminated by pesticides. Multiple chronic diseases, such as cancer and Parkinson’s disease, have been associated with pesticide exposure [9].

In terms of intentional self-poisoning, LMICs account for about 77% of the 700,000 annual deaths by suicide globally. The World Health Organization (WHO) reports pesticide self-poisoning as one of the most common means of suicide globally. It is estimated that 14–20% of suicides are due to pesticide ingestion [16], though numbers might be higher due to misclassification, underreporting and poor reporting systems [17]. LMICs are disproportionately affected by pesticide suicides. In countries in Asia, pesticides are one of the most common means of suicide, for example, in India, Nepal, China and Sri Lanka [16]. Pesticide poisoning is thus a significant clinical and public health challenge in LMICs. Individuals who have ingested pesticides need clinical treatment. In Sri Lanka, qualitative research on health care providers’ perceptions of treatment of pesticide poisoning found challenges related to lack of medication, equipment and staff. Furthermore, the staff highlighted how the mental health concerns behind a case of self-harm needed to be further addressed [18].

The most common pesticides used in LMICs are organophosphorus (OP) and carbamate insecticides such as parathion and carbosulfan [19,20]. These insecticides inhibit the enzyme acetylcholinesterase (AChE) in both target insects and exposed humans. This results in accumulation of the neurotransmitter acetylcholine and overstimulation of acetylcholine receptors, which can cause unconsciousness (coma), muscle weakness, difficulties with breathing, and ultimately, in severe cases, death [21]. The key treatment for this poisoning involves injecting a medical antidote called atropine [22]. This medicine blocks the effect of acetylcholine on its receptors and so acts to counter the poisoning. If given early enough and in sufficient doses, it can prevent many of the complications of poisoning.

However, a major problem identified by farming communities is the delay in receiving treatment. This can be for a variety of reasons, including distance or delays in transportation to hospital, delays in admission to hospital, and delays in receiving treatment once at the hospital [23–26]. Poisoning progresses while patients wait for treatment. As patients become unconscious, they lose control of the muscles around their airway and then may breathe in (aspirate) the contents of their stomach (food, gastric juice). This results in pneumonia which may kill patients even if they survive hospital presentations and good-quality medical care [27,28]. There is a need for a treatment that can be given in the village or field – very soon after the exposure and before transfer to hospital – and which can keep the patient well during transfer and while awaiting admission and treatment. Atropine autoinjectors, easy to use and portable devices for the injection of pesticide antidote, offer one possible such treatment.

Atropine auto-injector devices were originally developed to treat soldiers and civilians affected by OP nerve agents in conflict contexts, such as sarin and Novichok agents used in Syria and Salisbury [29]. They allow the atropine medicine (plus sometimes other antidotes such as pralidoxime, obidoxime and diazepam) to be rapidly administered by injection into the muscles of the thighs. Several atropine autoinjectors can be administered to the same patient, using different sites in the muscle. Experience and evidence indicate that no high-level training is needed to administer auto injector devices and civilians can be taught to use them safely [30–33]. They were, for example, distributed and stored at home in Israel at the peak of the Gulf War [34,35]. Atropine autoinjectors have a typical shelf life of more than 2 years, even in a hot country.

Each autoinjector device usually contains 2 mg of atropine, which is a common starting dose for intravenous injection into patients with OP or carbamate poisoning. Current military protocol suggests that three auto-injectors (6 mg) should be used for each patient [36]. The patients do not have to be ill when they are given the dose – it is usually given when there is a concern about exposure before the patient becomes ill.

These doses seem safe even in people who ultimately turn out not to be exposed to OP or carbamates. The best data are derived from studies of children during the Gulf War who accidentally injected themselves or who were injected with atropine autoinjectors [35]. Although some received doses as high as 17-fold more than standard doses for age, no deaths or serious injury were noted among 240 children who presented to hospital [37]. A further study of 142 children who injected themselves (or were injected by others) with a combination of atropine and trimedoxime showed no concerning adverse effects [35].

Atropine autoinjectors thus offer the potential opportunity to store an effective treatment near agricultural workers’ fields that could be reached within minutes by either the person or fellow villagers. Early intramuscular administration of atropine in the village could offer several hours of treatment that would markedly improve safety during the journey to hospital, reducing complications and saving lives.

However, it is not yet clear whether such an approach will be acceptable or feasible for community members in rural communities distant from health care. Our informal discussions with communities affected by pesticide poisoning indicates an interest in the idea of emergency care for poisoning in their communities, but there are many questions that need answering before formal trials and routine provision can be effective at saving lives. This includes whether auto injector devices can be introduced into, and accepted by, a rural village with minimal experience of such emergency health care? Who would store the auto-injectors and where? How would their use be managed, and who would replace them once used or expired? How do people anticipate the intervention might influence care-seeking and local power dynamics? Besides its implications for treatment, answering these questions would make a substantial contribution to social science research aiming to unfold the mechanisms behind pesticide poisoning in Sri Lanka and beyond.

The aim of this study is to undertake a situational analysis to understand how atropine autoinjectors might be best integrated into the social and spatial context of rural communities in Sri Lanka with high levels of pesticide use. The situation analysis will constitute the first phase of an implementation research trial introducing atropine autoinjectors in these and nearby communities. We will draw from the methods and theories of social science, and anthropology in particular. Anthropology is a discipline with great potential in contributing to implementation science in global health research but to date remains underutilised [38]. Specifically, this study intends to:

- explore the context of pesticide poisoning, including social, cultural and economic contexts and spatial and temporal dimensions of poisoning events;
- map current health care provision in the selected communities; and
- explore perceptions of community members’ and informal and formal health care providers around how atropine autoinjectors might be used in the community setting.

## Methods

### Study setting and context

The study will be conducted in the LMIC Sri Lanka. Sri Lanka is known for its successes regarding the implementation of primary health care, especially the use of community-based health services. This has included the staffing of rural health centres with midwives, who would be close to families and communities, and thereby reducing the need at the hospital-level [39]. In Sri Lanka, the majority of rural communities are involved in agriculture as their main livelihood, resulting in the common use of pesticides. Rates of pesticide poisoning are also high [40]. Self-poisoning is the most common method of non-fatal self-harm in individuals presenting to hospital; in 2019, pesticide self-poisoning accounted for 26% of all suicide deaths [(822 of 3135 suicide deaths) [41]].

Qualitative research has found that, in the Sri Lankan context, self-harm is often a means to communicate something that cannot be said in words in a society where hierarchy and positioning in society is important [42–44]. While it is sometimes described as a response to a trivial concern, in-depth research has found it to be a culmination of individuals living difficult lives, influenced by a range of daily stressors [42]. Marecek and Senadheera refer to the common form of self-poisoning as ‘dialogue suicide’ in contrast to ‘monologue suicide’, which is often seen in HICs [43]. Self-harm in Sri Lanka is often following a conflict with a significant other and has been found on occasions to be a response by women after arguments or physical abuse by their husbands to ‘teach him a lesson’ [42].

Specifically, the study will initially be conducted in three villages in the Anuradhapura District, North Central Province of Sri Lanka. New villages will be included until saturation is reached. The selected rural villages are located across the district and purposely selected based on: variation in farming practices and crops, high incidence of poisoning – both intentional and non-intentional (from existing unpublished data), logistics for research access, variable distance to the nearest health facilities, social capital, and our relationships with the communities.

### Research team

In Sri Lanka, this study is nested within the South Asian Clinical Toxicology Research Collaboration (SACTRC), based in Sri Lanka and with an international partnership of clinicians and researchers from Sri Lanka, Australia, UK, Germany, and Denmark (https://www.sactrc.org/). Since 2004, SACTRC has conducted research related to toxicology and pesticide poisoning, in close collaboration with multiple Sri Lankan partners, including the Sri Lankan Ministry of Health. The research team for this specific study is interdisciplinary with researchers proficient in toxicology, anthropology, participatory methods and community-based interventions from Sri Lanka, UK and Denmark. Furthermore, we will make use of a logistical set-up of research assistants already working in the study area.

### Study design

This project draws on qualitative methods to explore pesticide poisoning in selected communities and community members’ and community health workers’ perceptions of how atropine autoinjectors might be used in their villages. Subsequently, this will be followed by an exploration of the process of having atropine autoinjectors available in communities and how they are made use of, which will help establish how atropine autoinjectors might best be stored and used in rural communities using pesticides.

We will explore the context of pesticide poisoning, community members’ experiences of health care provision, and their perceptions of autoinjector devices and potential facilitators and barriers influencing the uptake of community-stored atropine autoinjectors. This research will contribute to a socially contextualised understanding of people’s experiences of pesticide poisoning and poisoning care in rural environments in Sri Lanka. This situational analysis will be used to develop the research design and protocol for the next phase of the study, which will explore and evaluate the implementation of community-based storage of auto-injectors in Sri Lankan villages.

### Sampling

We will obtain written permission from the Regional Director of Health Services and Divisional Secretaries of each DS division. The research team, consisting of a post doc, who has previously conducted community-based research in the area, as well as research assistants, will approach the selected communities for enrolment. The Grama Niladharis (GN), leaders for each village, have already been approached and verbally agreed to us conducting the study in their village. To obtain written consent from the GN, the research team will first meet with GNs and discuss the study. Here, researchers will explain the purpose of the study and how it will be useful for the community. Consent will be documented using a village-level consent template. The researchers will also explain what taking part in the study will involve, both for the community and for the individuals, as well as the potential risks. The research team will then identify relevant individuals and invite them to participate in the study face-to-face. The research team will provide each participant with a participant information sheet at this time.

### Research methods

#### Community-level situational analysis

To obtain an understanding of the context of pesticide poisoning in the three villages and to explore hospital-based health care professionals’ (objective 2), and community members’ and community health workers’ perceptions of atropine autoinjectors and potential facilitators and barriers influencing the uptake of community-stored atropine autoinjectors (objective 3), we will make use of semi-structured interviews, ethnographic observations, oral history interviews, participatory mapping, Focus Group Discussions (FGDs) as well as existing secondary data sets.

##### Oral-history interviews

In-depth oral history interviews will be carried out with individuals from households that have experienced intentional or non-intentional pesticide poisoning (either the individual who consumed poison or a family member) to gain an understanding of the spatial and temporal dimensions of pesticide poisoning events, and experiences of health care and treatment. Interviews will follow a topic guide and take a chronological structure, exploring the temporal unfolding of specific poisoning events, and the place of those events within the participant’s biography. The number of participants will depend on the available number of eligible participants in each village. Participants will be selected based on secondary baseline survey data shared from an associated study (see below). Approximately 5 interviews will be conducted in each village (*n* = 15). Interviews will be carried out by a post doc trained in anthropology, as well as research assistants, following a topic guide. Semi-structured interviews will be recorded where permission is given, and notes will be taken during and directly after the interview.

##### Semi-structured interviews

In-depth semi-structured interviews will be carried out with the community members to explore their thoughts, beliefs, experiences and perceptions in relation to introducing atropine autoinjectors in their specific village. Especially, we will explore where it should be stored and who should administer it. Thus, the aim is to gain a deeper understanding of the social, cultural and economic factors that underlie community practices and interactions, important for identifying the priorities of community members. Some of these may be carried out as follow-up interviews with the same community members as the oral histories. In this case, participants will only be asked to sign one consent form if they do both. Depending on saturation, approximately 5 interviews will be conducted in each village (*n* = 15), by a post doc trained in anthropology, as well as research assistants, following a topic guide (see annex). Furthermore, about five interviews will be conducted with community health workers working in the community and at nearby local hospitals/clinics (*n* = 5). We will use physical examples of (disabled) atropine autoinjectors as a prompt for discussion. Furthermore, a stakeholder analysis will be conducted to explore pathways to care for pesticide poisoning in the rural Sri Lankan context. Specifically, it will explore where potential delays in treatment occur, i.e. getting from the community to a hospital, within the hospital, or in the referral from one hospital to another. Semi-structured interviews will be conducted with health care providers in referral hospitals, district health managers, and community health workers (*n* = 10). Semi-structured interviews will be recorded where permission is given, and notes will be taken during and directly after the interview. When translation is needed, the interviews will be thoroughly discussed after they have been conducted. Participants may be asked for a follow-up interview after initial analysis.

##### Ethnographic observation

Ethnographic observation will be used to gain an understanding of the socio-spatial organisation, everyday routines and interactions in the three villages and the social, economic and cultural context of pesticide poisoning, including an understanding of agricultural livelihoods, kinship and politics. Ethnographic observations will be undertaken alongside the interviews, participatory mapping and FGDs to document the context for those interactions. A post-doc trained in ethnographic methods, assisted by field assistants, will spend approximately 1 month conducting ethnographic observations in one village, which will be selected after some of interviews, have been completed on the basis of the willingness of the community and logistics.

##### Participatory mapping

To ensure the priorities of the study population are central to the study and its approach, we will make use of participatory mapping. Participatory mapping is based on the premise that local communities possess expert knowledge about their local environments. It has previously been used to explore interests by diverse groups, such as farmers and indigenous people [45]. We will not use formal cartographic techniques and will instead employ basic drawing materials to collaborate with participants and build maps of (i) the local area, (ii) the relationship between specific sites/spaces and pesticide poisoning risks/events and (iii) the local health system and routes to care. Participatory mapping will take place within households, and all adult members of the household will be invited to contribute collectively to the process. Approximately 5 households will be selected from each village (*n* = 15) using convenience or snowball sampling while ensuring variety in terms of age, gender, livelihood and household income, thereby capturing different perspectives and concerns.

##### Focus group discussions

To understand how atropine autoinjectors might best be stored, and used in rural communities using pesticides, FGDs will be conducted with community members in the selected villages. The FGDs will focus on group norms and participants’ beliefs, thoughts, experiences and perceptions, allowing for the collection of data that participants may be more likely to reveal in a social and interactive setting than in one-to-one interviews. The FGDs will also reveal the diversity of views in a particular sub-group and are useful for determining differences of opinion and normative positions [46]. The aim is thus to understand community members’ view on atropine autoinjectors being stored locally, how they value them as an addition to the health care offered to individuals poisoned by pesticides, and how (where and by whom) the autoinjectors might be used. A starting point for discussion will be the maps generated during the participatory mapping. Furthermore, a key focus will be on identifying one or more relevant focal point(s) in each village who will be responsible for handling the atropine autoinjectors. Two FGDs will be conducted in each village with 6–12 participants in each (*n* = 15) in gender segregated groups. Participants will be purposely selected from male and female community-members and across age-groups, including those who do and do not use pesticides or work in the agricultural fields. Village leaders will be included in one of the FGDs for each village – both as part of initial data collection and after initial data analysis to ensure that the identified analytical themes resonate with them. Group discussions will be co-facilitated by two members of the research team using a topic guide (see annex), voice recorded (if permission granted), transcribed and translated.

#### Secondary data

As part of a data-sharing arrangement, we will have access to data from an associated quantitative study in similar villages in the North Central Province. This will include data from a baseline survey of participants to assess the history of occupational pesticide poisoning and regular 2-month follow-up to monitor if there is any acute pesticide poisoning, which will be carried out between December 2023 and November 2025. This data will be used to enhance the situational analysis through an understanding of the spatial, temporal and demographic distribution of pesticide poisoning events in those villages and will help us to identify participants for the oral history interviews.

#### Hospital-level situational analysis

A hospital-level situational analysis will be conducted to understand the infrastructural context, intra-hospital social relations, perceptions towards poisoning within the hospital setting and patient treatment pathways that influence the treatment of patients presenting with poisoning and the potential impact of autoinjectors on care pathways. This will be carried out by a postdoc with experience conducting hospital ethnographies, assisted by a research assistant, and will consist of interviews and participatory mapping with health staff, and observations of the medical wards or emergency departments in secondary-care hospitals in which patients presenting with poisoning are treated.

Interviews will be conducted with hospital administrators (*n* = 2), doctors (senior *n* = 2, junior *n* = 3) and nurses (*n* = 3) working in the emergency ward. These interviews will explore participants’ perceptions related to poisoning through pesticide ingestion and experiences treating patients presenting with poisoning through pesticide ingestion in the hospital. Participatory mapping will be carried out as part of the interview process to understand participant perceptions and experiences of patient care-pathways and the possible impact of community-based auto-injectors.

### Data analysis

Preliminary qualitative data (from interviews, mappings, focus group discussions, and ethnographic field notes) will be analyzed in the field using deductive primary and secondary codes developed from the interview guides, leading to refinement of the data-collection tools and further sampling till saturation has been reached. Codes will be refined in regular team meetings and inductive codes added as relevant. In the post data-collection period, NVivo software will be used to code interview and FGD transcripts. Meta-level themes (implicit topics that organise groups of repeating ideas) will begin to emerge from this coding process. These themes will be: (1) shared with community leaders in each village to ensure that they resonate with them, (2) refined during a workshop conducted in Sri Lanka with the entire research team. Based on outcomes of this workshop, we will put the refined themes into dialogue with the existing literature, leading to the development of new understandings and concepts for understanding pesticide poisoning, and the provision of emergency health care in the community.

### Ethical considerations

The study will be conducted in accordance with the principles of the International Conference on Harmonisation Tripartite Guideline for Good Clinical Practice (ICH GCP) in addition to the principles of the ethics committee/Institutional Review Boards (IRBs) who have reviewed and approved this study. Approvals have been obtained from the University of Edinburgh Medical School Research Ethics Committee (23-EMREC-039) and from Ethics Review Committee, Faculty of Medicine and Allied Sciences, Rajarata University of Sri Lanka (ERC/2023/4).

Access to villages will be obtained through village leaders (GN) and the study will be presented at village meetings, such as funeral society meetings. FGDs and community engagement activities will be planned in advance with GNs and other village leaders. It is of utmost relevance to the research team to ensure that participants are met in an open and non-judgemental manner in a safe environment. This is particularly relevant during the oral history interviews with individuals from households that have experienced intentional or non-intentional pesticide poisoning (either the individual who consumed poison or a family member), and particular attention will be given to ensure that they are comfortable taking part in the study. Written informed consent will be sought from all participating individuals (?) and they will be informed and reminded that they can withdraw from the study at all times. All data will be anonymous, leaving out participant names and addresses.

Dissemination of findings will be done through publications in peer-reviewed open access journals, and sharing with Sri Lankan and international stakeholders through updates and blog-posts at the study website (include), and through popular media when possible.

## Discussion

Although their negative health impacts are no mystery, pesticides are a fact of daily life for agricultural workers throughout the world. Acute poisoning stemming from both intentional and unintentional pesticide exposure disproportionately impacts farmers in LMICs [47]. Our study explores the possibility of introducing a simple, technical intervention to improve care for acute pesticide poisoning in Sri Lanka. While promising, such interventions shape, are shaped by, and depend on the social and cultural contexts in which they are embedded. The situation analysis presented in this paper therefore aims to unearth the social and cultural conditions in which the intervention will be introduced, including health systems infrastructures and relationships, and explore the perceptions of the planned intervention among those who are expected to benefit from it in Sri Lanka, thereby ensuring the best possible foundation for an intervention.

Indeed, this study is quite unique in leveraging an anthropological approach to implementation science, which has gained traction in global health fields of practice over the past two decades. Implementation science provides an important complement to outcome- and impact-oriented assessments, which have long been dominant in global health practice [48,49]. Anthropological methods and theories are particularly well suited for understanding the power dynamics, structural and social dimensions, and unanticipated consequences of interventions, but have thus far been underleveraged in implementation science [38,50]. This study draws from an explicit engagement with anthropological methods and theory and will provide insights on how anthropology can more fully benefit implementation science.

Atropine autoinjectors offer a promising technical solution to improve care for intentional and unintentional acute pesticide poisoning, an urgent and neglected public health issue in Sri Lanka and throughout the Global South. Proposing autoinjectors as a technical solution to a health issue reflects, in many ways, global health aspirations which seek to harness the power of technology and innovation to address global health issues and promote health equity [51–53]. Often, such solutions are proposed as autonomous tools which promise to circumvent challenging social conditions and under-resourced health systems [54,p.270, 55]. However, a large volume of literature from the social sciences has demonstrated that technological fixes are always shaped by and rely on the social and relationship contexts in which they are introduced [56–58]. Our situation analysis recognises the value of social science scholarship and theory for informing the introduction of technologies to address global health issues, and specifically, applies a socially informed approach to the introduction of atropine autoinjectors in Sri Lanka.

In addition to informing the planned intervention, this research poses several broader social science questions relevant to global health. As mentioned, biomedical devices are increasingly proposed in global health fields of practice for decentralisation and strengthening of health service provision in LMICs [53]. While this reflects predominant value orientations in global health, anticipations of technical devices in settings in which they are intended for introduction remains little explored. This study will help us understand the meanings and values community members and health care workers ascribe to the autoinjectors before their introduction, and how these are shaped by wider social constructions of biomedicine and biomedical technologies and experiences of health systems. Specifically, we aim to find answers to the following questions: How do people imagine proposed ‘technological fixes’ to health issues in relation to the formal health system and how is this shaped by their perceptions of and relationships with the health system? How do established understandings and perceptions of pesticide poisoning inform people’s hopes, aspirations and expectations of technologies? Illuminating the anticipations of people expected to benefit from technologies can contribute to more nuanced discourses in global health fields of practice, recognising compatible and competing value orientations.

This research will also contribute to an increased understanding of the health systems in Sri Lanka in relation to health seeking behaviour in emergencies, relationships between people and health systems, and possibilities for extending the health system into communities through technologies such as atropine autoinjectors. Even as health systems strengthening discourses gain ground in global health policy and programming discourses, we have also seen increased attention to our limits on understanding the boundaries of health systems and how they function [59]. This study will contribute to a qualitative understanding of health systems as spatially and temporally situated, and examine how they are navigated for emergency care, particularly related to acute pesticide poisoning.

This qualitative research builds on and adds to a strong tradition of social science research in Sri Lanka. This includes Obeyesekere’s seminal research on moral regulation of behaviour [60,61] and Tudor Silva’s extensive work on inequality, caste, ethnicity and health in Sri Lanka [see for example, 62–64]. Social science research has also been conducted with a specific focus on self-harm, including pesticide poisoning [65,66], explorations of the meanings of poison [67–69], and the social determinants of self-harm, such as gender roles [70–72] kinship [44], and the harmful use of alcohol [42].

As we engage with and build on these rich investigations, the timing of our study is relevant. Sri Lanka has faced numerous challenges in recent years, including terrorist attacks, a pandemic and political uncertainty, which have all heavily influenced daily life. Furthermore, climate change is a real challenge in Sri Lanka, with the country experiencing one of the warmest years on record in 2023 and continuously ranking high on the Global Climate Risk Index. At the same time, the country has recently experienced heavy rainfall compared to previous years. Both could have far-reaching implications for the agricultural sector, especially in the Northern Region and the Dry Zone of Sri Lanka, where this study will be conducted [73,74]. For example, food security at both national and household levels are expected to be influenced by changes in the climate [75]. In addition, several far-reaching political decisions have been implemented impacting the availability of pesticides, including bans of the most toxic pesticide for humans. In May 2021, the Sri Lankan government introduced an ‘overnight’ national ban on the import of all synthetic agrochemicals i.e. fertilizers and pesticides. The sudden political decision left the country’s farmers unsure about whether and how to rapidly shift from conventional agriculture to organic agriculture. The policy decision led to a marked fall in crop production, forcing the country to again import staple foods, sending prices soaring. Therefore, the ban was revoked in November 2021. It is within this new reality that our research will expand our understandings of village life, including perceptions of pesticide use, in rural Sri Lanka.

Finally, this study reflects our ethical commitment to carrying out research with communities, rather than on communities. Community participation is now recognised as fundamental to ethical global health practice, and a requirement of good research among some donors, such as the UK NIHR who is funding the project. Community members may be wary of new interventions, particularly those introducing new technologies such as atropine autoinjectors. These involve intrusion into the body, and intimate and embodied work of community members not formally trained as health workers.

Some social science scholars have illuminated that community engagement does not always result in reflecting the interests and desires of communities. Paradoxically, such efforts towards engagement can be instrumentalized and used to serve programmers’ purposes rather than communities or reflect and reinforce local power dynamics [76,77]. This situation analysis reflects our commitment to carry out work with communities that respond to their interests and preferences. To ensure this from a programming perspective, we have built sufficient flexibility into our planning to respond to community inputs.

## Conclusion

Acute pesticide poisoning disproportionately affects the most vulnerable farmers in LMICs. Atropine autoinjectors present a promising device that can be managed by and used in communities to extend biomedical care into health system peripheries and improve health care of those needing urgent attention. However, its success will hinge on the social and cultural conditions in which it is embedded. The current situation analysis seeks to unveil these conditions to design a socially informed approach to implementation. Additionally, it will generate new knowledge and understanding of social life in a rapidly shifting Sri Lanka, on important questions in global health policy and programming, and promote active engagement of the community.
